# L-Histidine resensitizes multidrug-resistant bacteria to carbapenems by inhibiting New Delhi metallo-β-lactamase activity

**DOI:** 10.1371/journal.ppat.1014728

**Published:** 2026-10-26

**Authors:** Yanqing Yin, Sulan Hu, Dan Fang, Yanhu Huang, Miaomiao Huang, Suhong Liu, Zhiqiang Wang, Yuan Liu

**Affiliations:** 1 Jiangsu Co-innovation Center for Prevention and Control of Important Animal Infectious Diseases and Zoonoses, College of Veterinary Medicine, Yangzhou University, Yangzhou, China; 2 Joint International Research Laboratory of Agriculture and Agri-Product Safety, the Ministry of Education of China, Yangzhou University, Yangzhou, China; 3 Institute of Comparative Medicine, Yangzhou University, Yangzhou, China; Shanghai Jiao Tong University Affiliated Chest Hospital, CHINA

## Abstract

Plasmid-mediated New Delhi metallo-β-lactamase (NDM)-producing Gram-negative pathogens have severely undermined the clinical utility of carbapenems, the last-resort antibiotics for multidrug-resistant infections. Given the limited pipeline of novel antibiotics, there is an urgent need for more effective anti-infective strategies. Through untargeted metabolomics and functional screening, we find that L-histidine (L-His), a key downregulated metabolite in NDM-positive bacteria, remarkably restores carbapenem susceptibility. Mechanistically, L‑His acts through two complementary pathways: (i) direct competitive inhibition of NDM enzymatic activity via zinc chelation at the active site, and (ii) upregulation of the nickel ion transport gene cluster *nikABCDE*, which increases intracellular Ni^2+^ accumulation and further suppresses NDM function through non‑competitive inhibition. Overexpression of *nikA* potentiates the bactericidal activity of meropenem, whereas deletion of *nikAB* attenuates the synergistic effect of L‑His and meropenem. In a murine peritonitis‑sepsis model, co‑administration of L‑His with meropenem significantly improves survival and reduces bacterial burden, with a favorable safety profile. Collectively, our findings reveal L‑His as a multi‑mechanistic metabolic adjuvant that combines direct enzyme inhibition with disruption of metal homeostasis, positioning it as a promising therapeutic strategy against NDM‑positive carbapenem‑resistant infections.

## Author summary

The rise of carbapenem-resistant bacteria, particularly those producing NDM enzymes, has left clinicians with few effective treatment options for severe Gram-negative bacterial infections. In this study, we show that L-histidine (L-His), a naturally occurring amino acid commonly used as a dietary supplement, can restore the killing of meropenem against NDM-producing superbugs. L-His works by competitively binding to the zinc ions in the NDM enzyme’s active site and disrupting intracellular Ni^2+^ homeostasis to suppress NDM activity via a non‑competitive mechanism. In both in *vitro* and in *vivo* infection models, L-His supplementation significantly enhanced bacterial clearance by meropenem and improved survival, without causing detectable toxicity. While more studies are needed, our findings suggest a promising approach to rejuvenate existing antibiotics with a simple, safe, and natural metabolic adjuvant.

## Introduction

The discovery and application of antibiotics have revolutionized modern medicine, drastically reducing mortality from bacterial infections. However, the indiscriminate use of these life-saving drugs has fueled a global antimicrobial resistance (AMR) crisis, one of the most pressing challenges to public health [1]. The horizontal transfer of antibiotic resistance genes (ARGs) has exacerbated the situation, with alarming projections indicating that by 2050, AMR could kill up to 10 million lives annually worldwide [2]. Carbapenem antibiotics, such as meropenem (MEM) and imipenem, represent a crucial class of β-lactam antibiotics used to treat severe nosocomial and complex abdominal infections [3]. These agents have long been considered a last-resort option due to their broad spectrum and high efficacy. However, the emergence of metallo-β-lactamases (MBLs) has severely compromised the effectiveness of carbapenems [4]. MBLs, including imipenemases (IMP), Verona integron-encoded metallo-β-lactamases (VIM), and the more recently identified New Delhi metallo-β-lactamases (NDM), are Zn^2+^-containing enzymes that catalyze the hydrolysis of the β-lactam ring, rendering bacteria resistant to all currently available bicyclic β-lactams, including carbapenems [5,6]. The U.S. Centers for Disease Control and Prevention (CDC) has classified carbapenem-resistant Enterobacteriaceae (CRE) as one of the most urgent threats, with nearly 50% mortality rates among hospitalized patients with bloodstream infections caused by these bacteria [7]. Since its initial detection in Sweden in 2009, NDM-1-positive bacteria have spread rapidly to over 70 countries [8]^-^ [9]. Plasmid-carried *bla*_NDM-1_ is highly transferable among major human pathogens like *Enterobacteriaceae*, *Pseudomonas* spp., and *Fusobacterium* spp [10]., and often co-harbors with other resistance determinants, giving rise to pan-drug-resistant superbugs [11,12]. Currently, the therapeutic arsenal against these pathogens is extremely limited [13]. Therefore, it is imperative to develop novel strategies to restore the efficacy of carbapenem antibiotics.

Accumulating evidence positions bacterial metabolism as a pivotal determinant of antibiotic susceptibility and resistance [14]. Metabolic states can modulate the activity of antibiotics. For example, a few high-resistance mutants may protect the survival of bacterial subpopulations by stimulating drug efflux pumps and oxidative stress protection mechanisms [15]. Furthermore, perturbations to bacterial respiration can significantly alter antibiotic killing activity, highlighting a profound link between metabolic activity and treatment outcomes [16]. This principle of “metabolic potentiation” opens promising avenues for adjuvant development. Consistently, our recent findings imply that exogenous indole-3-acetic acid improves antibiotic efficacy in eliminating persisters [17], a subgroup of bacterial cells that can survive transitory antibiotic exposure, by activating bacterial metabolic pathways. Deoxycholic acid has also been shown to improve antibiotic efficacy by increasing antibiotic accumulation and modulating the oxidant-antioxidant system [18]. Notably, we recently revealed that L-methionine supplementation reverses high-level tigecycline resistance by triggering DNA methylation [19]. In addition, carbon sources and some amino acids can potentiate antibiotic activity by boosting the proton motive force (PMF) [20–22]. These findings collectively underscore the therapeutic potential of targeting bacterial metabolism to resensitize drug-resistant pathogens.

In this study, we employed a metabolomics approach to identify metabolic disparities between NDM-negative and -positive bacteria. Notably, we showed that L-histidine (L-His) acts as a potent adjuvant that reverses the antibiotic resistance phenotype of NDM-positive bacteria and prevents the development of resistance. We confirmed the therapeutic efficacy of L-His and MEM combination in a murine peritonitis model. Mechanistically, L-His directly inhibits NDM enzyme activity by specifically binding to zinc ions in its active site. Meanwhile, L-His upregulates the expression of Ni^2+^ transporter genes, facilitating Ni^2+^ uptake that further inhibits NDM activity through a non-competitive mechanism. Overall, our work reveals a unique, multi-faceted strategy wherein L-His restores MEM efficacy against NDM-positive bacteria, positioning it as a promising, metabolism-targeting adjuvant in the fight against antimicrobial resistance.

## Results

### Metabolomic analysis between NDM-negative and -positive bacteria

To decipher the metabolic alterations associated with NDM carriage, we conducted a non-targeted metabolomics analysis comparing engineered *E. coli* DH5α (pUC19) (NDM-negative) with *E. coli* DH5α (pUC19-*bla*_NDM-5_) (NDM-positive). Principal component analysis (PCA) of the two groups of samples showed that the variance contribution of principal component 1 was 50.90% and that of principal component 2 was 12.20%, which could better reflect the characteristics of the data, and the two groups of samples were separated along the direction of PC1, indicating that the metabolic profiles differed significantly between groups (Fig 1A). Among the 199 metabolites detected, 20 were significantly up-regulated and 38 were down-regulated in NDM-positive strains relative to the NDM-negative bacteria, while 141 metabolites showed no notable changes (Fig 1B). We further visualized the 58 differential metabolites using a circle clustering heatmap (Fig 1C). KEGG-based categorization indicated that amino acids constituted the largest class of differential metabolites (Fig 1D), a finding corroborated by pathway analysis, which identified amino acid metabolism as the most prominently affected category (Fig 1E). The systemic relationships among these metabolites and pathways were further visualized in a KEGG enrichment analysis network diagram (S1 Fig). Among the key changes, a pronounced downregulation of L-histidine (L-His) was observed, suggesting its potential role in metabolic reprogramming.

**Fig 1:**
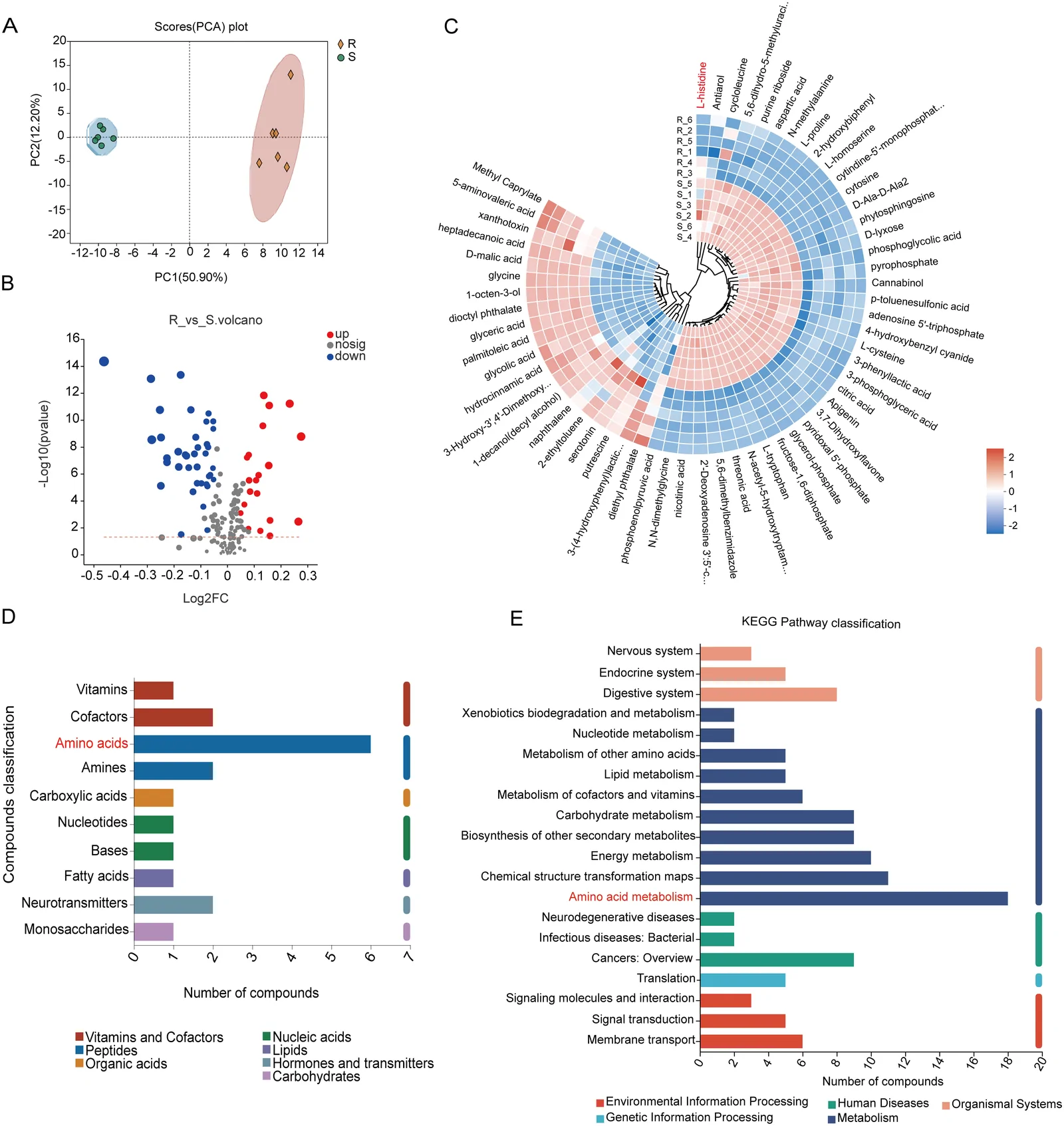
Non-targeted metabolomic profiling of NDM-negative and -positive bacteria. **(A)** PCA of metabolomics. An unsupervised PCA plot was applied to examine the clustering characteristics of different metabolites. Each colored dot represents an individual biological sample (n = 6). **(B)** Volcano plot of differentially expressed metabolites (DEMs) annotated for the NDM-positive bacteria versus NDM-negative bacteria; log_2_FC was calculated to account for the direction of abundance difference. Metabolites with log_10_FDR values ≥ 1 and log_2_FC ≥ 1 or log_2_FC ≤ -1 were considered significantly different (red: up-regulated; blue: down-regulated; grey: non-significant). **(C)** Circle clustering heatmap of the 58 DEMs. The outer ring indicates metabolite regulation status (red: up-regulated; blue: down-regulated) in the NDM-positive bacteria, with L-histidine highlighted. **(D)** KEGG chemical categorization of the DEMs. The amino acid category was predominant and was highlighted in red. **(E)** KEGG pathway classification of the DEMs, showing amino acid metabolism as the most significantly enriched category.

### L-Histidine potentiates meropenem against NDM-positive bacteria

Untargeted metabolomic profiling revealed substantial amino acid consumption and pathway remodeling in NDM-positive engineered strains. Consistent with this metabolic signature, targeted quantification in clinical isolates confirmed a significant reduction in intracellular L-His abundance (S2A Fig). Therefore, we hypothesized that supplementing key low-abundance metabolites, critical to their regulatory networks, could reprogram metabolism to mirror NDM-negative strains, potentially reversing resistance and restoring antibiotic susceptibility. To investigate this hypothesis, we screened the above-mentioned amino acids, and examined survival of clinically isolated NDM-positive bacteria under sub-inhibitory concentrations of MEM with the addition of different amino acids. Remarkably, only L-His could significantly potentiate the bactericidal activity of MEM, reducing bacterial viability by more than 3-log_10_ CFU (S2B Fig). We further evaluated the potentiation spectrum of L-His against other β-lactam antibiotics, including cefotaxime (CTX), ceftazidime (CAZ), ceftriaxone (CRO), imipenem (IPM), amoxicillin (AMX), ampicillin (AMP), ticarcillin (TIC), piperacillin (PIP), and cloxacillin (CLO). With the exception of AMP, all tested antibiotics exhibited noticeable synergistic bactericidal activity with L-His, among which MEM showed the strongest effect (S2C Fig).

We then quantitatively assessed the interaction between L-His and MEM using a standard checkerboard microdilution assay. Interestingly, the combination demonstrated robust synergistic activity against NDM-positive bacteria, with a fractional inhibitory concentration index (FICI) of 0.1875 (Fig 2A). In contrast, no synergistic or additive effect was observed in the NDM-negative *E. coli* ATCC 25922 (Fig 2B), indicating that the potentiation is specifically linked to the presence of NDM. This specificity was further supported by the consistent synergistic effect observed in engineered NDM-1-positive *E. coli* MG1655, clinical isolates of NDM-1-positive *E. coli* C3, along with NDM-5-positive *E. coli* G6 and *Pseudomonas* HNCF44W (Fig 2C). While L-His alone had negligible effects on bacterial growth, its combination with MEM resulted in significant growth inhibition across all strains tested (S2D–S2F Fig). Notably, the addition of L-His reduced the MICs of MEM against these NDM-positive strains, both clinical isolates and engineered strains, by 1- to 32-fold (S1 Table), further supporting the synergistic potential of this combination. Time-kill assays confirmed a time-dependent bactericidal effect of the L-His and MEM combination. Treatment with MEM (1/2 MIC) plus L-His (10 mM) for 48 h reduced bacterial counts by more than 4-log_10_ compared to the untreated or MEM-only controls (Fig 2D-F). This synergistic bactericidal action was dose-dependently amplified as L-His or MEM concentrations increased (Fig 2G, H and S2G Fig–S2I Fig).

**Fig 2:**
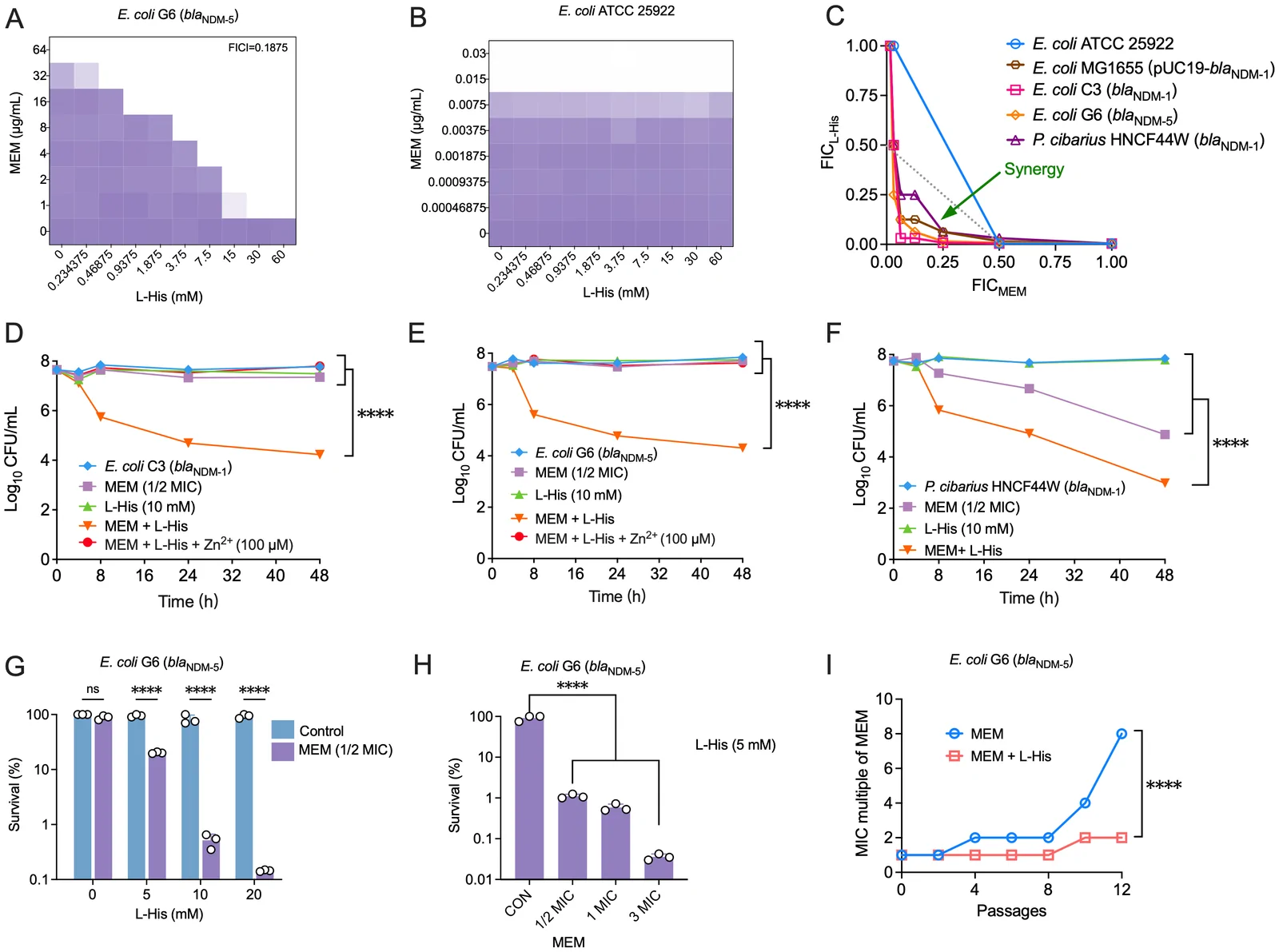
L-Histidine restores MEM susceptibility against NDM-positive bacteria. **(A and B)** Checkerboard microdilution assays of L-His and MEM combination against NDM-positive (A) and NDM-negative (B) bacteria. **(C)** Equivalence line plot of L-His and MEM combination against multiple NDM-positive strains. The dashed green line represents the ideal equivalency line. The data points below the line denote synergistic effects. **(D-F)** Time-kill curves of MEM (1/2 MIC) with or without L-His (10 mM) against three NDM-positive strains, including *E. coli* C3 (*bla*_NDM-1_, **D**), *E. coli* G6 (*bla*_NDM-5_, **E**) and *P. cibarius* HNCF44W (*bla*_NDM-1_, **F**). **(G, H)** Dose-dependent bactericidal effects of L-His and MEM combination against *E. coli* G6. **(I)** Development of MEM resistance in *E. coli* G6 during serial passage with sub-MIC MEM (1/4 MIC) in the presence or absence of L-His (10 mM). Experiments were performed with three biological replicates and data are mean ± SD, and the statistical significance was determined by one-way ANOVA or unpaired *t*-test. ***P < 0.001, ****P < 0.0001. ns, not significant.

To investigate whether L-His influences the evolution of MEM resistance, we performed serial passage experiments with *E. coli* G6 under sub-MIC MEM pressure in the presence or absence of L-His. Strikingly, the addition of L-His substantially suppressed the emergence of high-level MEM resistance over 12 passages (Fig 2I). Taken together, these findings indicate that L-His acts as a potent and specific synergist for MEM against diverse NDM-positive bacteria, and may impede the development of carbapenem resistance.

### L-Histidine suppresses the hydrolytic activity of NDM

The synergy between L-His and MEM, specific to NDM-positive strains, prompted the hypothesis that L-His directly inhibits NDM activity. To test this, we first examined the interaction between L-His and NDM (PDB ID: 4TZE) by molecular docking. L-His readily occupied the catalytic pocket of NDM-5, forming hydrogen bonds with the two zinc ions in the active site (Fig 3A). Consistently, an in *vitro* enzyme inhibition assay confirmed that L-His substantially reduced the hydrolytic activity of NDM-5 (Fig 3B), and enzyme kinetic analysis revealed a competitive inhibition mode (Fig 3C). Given the essential role of the dizinc center in NDM catalysis, we reasoned that L-His might competitively target the active-site zinc ions. Indeed, the inhibitory effect of L-His on NDM-5 was progressively reversed by increasing concentrations of exogenous zinc ions (Fig 3D), demonstrating that the inhibition is zinc-competitive and reversible. Furthermore, we tested the L-His and MEM combination against clinical isolates producing IMP (class B metallo-β-lactamase) or KPC (class A serine β-lactamase). Synergy was observed exclusively in the IMP-producing strain, not in the KPC producer (S3 Fig), consistent with a zinc-chelation mechanism that specifically targets class B metallo-enzymes.

**Fig 3:**
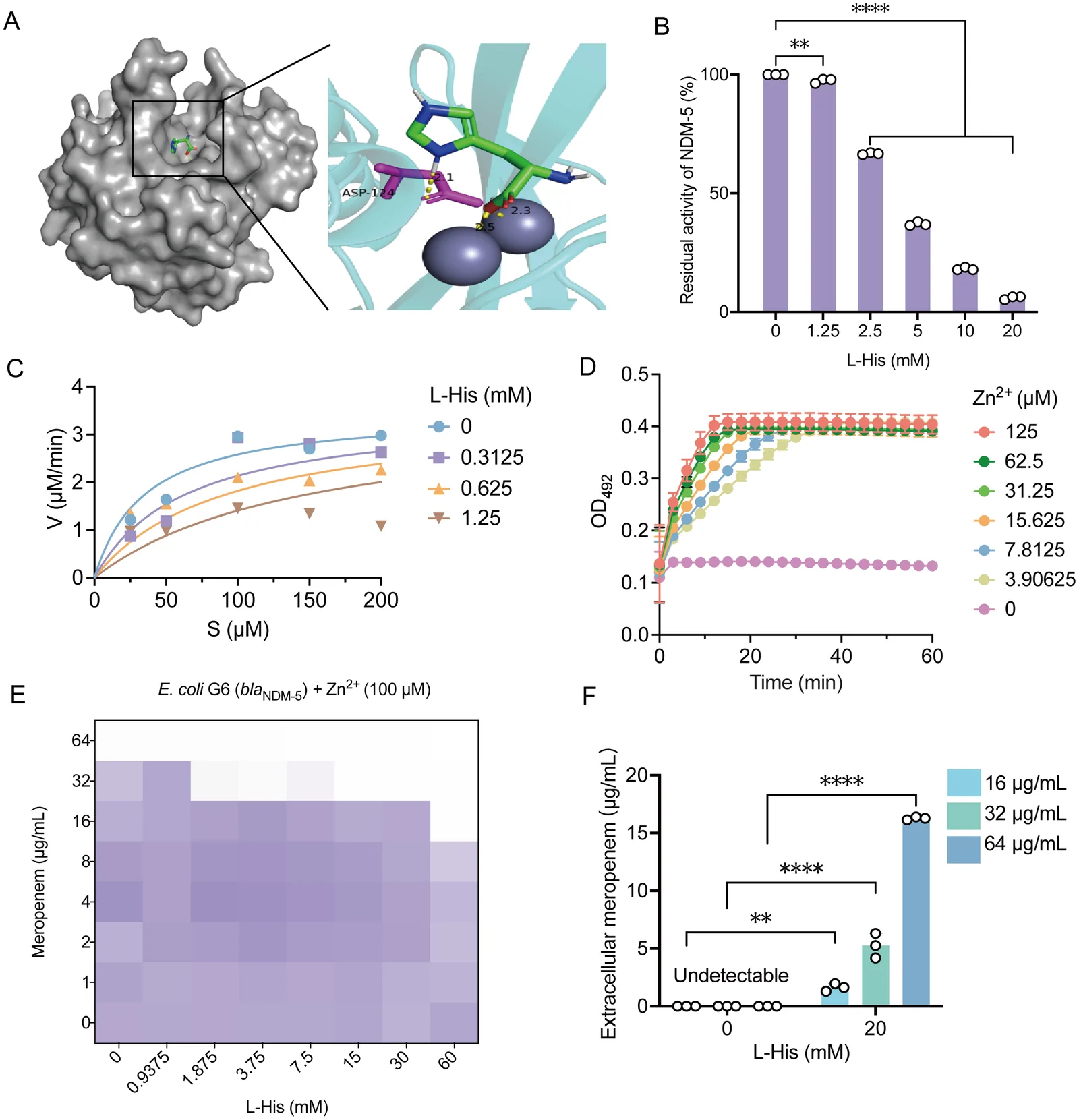
L-Histidine directly inhibits the enzymatic activity of NDM. **(A)** Molecular docking analysis of L-His into the NDM-5 active site using AutoDock Vina and visualized with PyMOL. L-His coordinates the two zinc ions in the catalytic pocket. **(B)** Effect of different concentrations of L-His on the hydrolytic activity of NDM-5 against cephalosporin nitrothiophene. **(C)** Nonlinear regression analysis of enzyme kinetics with L-His ranging from 0 to 1.25 mM. The initial reaction velocity (V) was plotted against substrate concentration (S). Data points were fitted (solid lines) to the Michaelis-Menten equation for competitive inhibition. The derived kinetic parameters are: Km, 35.01 μM; Vmax, 3.482 μM/s; and Ki, 0.3882 μM. The unchanged Vmax and increased apparent Km indicate competitive inhibition. **(D)** Recovery of NDM-5 (0.1 μM) activity inhibited by L-His (10 mM) upon addition of different concentrations of exogenous Zn^2+^ for 1 h. **(E)** Microdilution checkerboard assay of synergy between L-His and MEM in medium supplemented with 100 µM Zn^2+^. **(F)** Extracellular MEM levels in the presence or absence of 20 mM L-His using liquid chromatography. The data from triplicate biological replicates were presented as mean ± SD, and the significance was determined by non-parametric one-way ANOVA. **P < 0.01, ****P < 0.0001.

We next verified the functional relevance of zinc competition in bacterial cells. In checkerboard assays performed with medium supplemented with 100 µM Zn^2+^, the synergistic effect between L-His and MEM was substantially attenuated (Fig 3E), and time-kill curves confirmed that exogenous zinc ions abolished the synergistic bactericidal activity of the combination (Fig 2D, E). These results demonstrated that L-His suppresses NDM activity by chelating the catalytic zinc ions, thereby protecting MEM from hydrolysis. To directly test this, we monitored extracellular MEM levels by LC-MS. Without L-His, MEM was rapidly degraded and fell below the detection limit even at a high initial concentration (64 µg/mL). Strikingly, the addition of 20 mM L-His dramatically preserved extracellular MEM levels (Fig 3F). Collectively, these findings establish that L-His acts as a direct, competitive, and zinc-sensitive inhibitor of NDM, effectively shielding MEM from hydrolysis and restoring its antibacterial activity.

### Activation of nickel transport contributes to L‑His‑mediated potentiation of MEM

While L-His has been shown to directly inhibit NDM to enhance the bactericidal effect of MEM, its potential transcriptional regulatory role in mediating synergistic antibacterial effects remained unclear. Therefore, to systematically address this, we conducted a comprehensive analysis of the transcriptomic changes in *E. coli* G6 treated with or without L-His (10 mM). PCA showed clear separation between the two groups (S4 Fig), with 67 genes significantly up-regulated and 90 genes down-regulated (Fig 4A). GO enrichment analysis revealed that the differentially expressed genes (DEGs) were significantly enriched in nickel-related transport processes, including nickel cation import across the plasma membrane, nickel cation transport, and nickel cation transmembrane transport (Fig 4B). KEGG enrichment analysis identified ABC transporters as the most significantly enriched pathway, with Sulfur relay system exhibiting the highest enrichment ratio (Fig 4C). Enrichment visualizations further illustrated that Ni^2+^ transport-related genes (e.g., *nikABCDE*) were among the most prominently up-regulated (Fig 4D, E).

**Fig 4:**
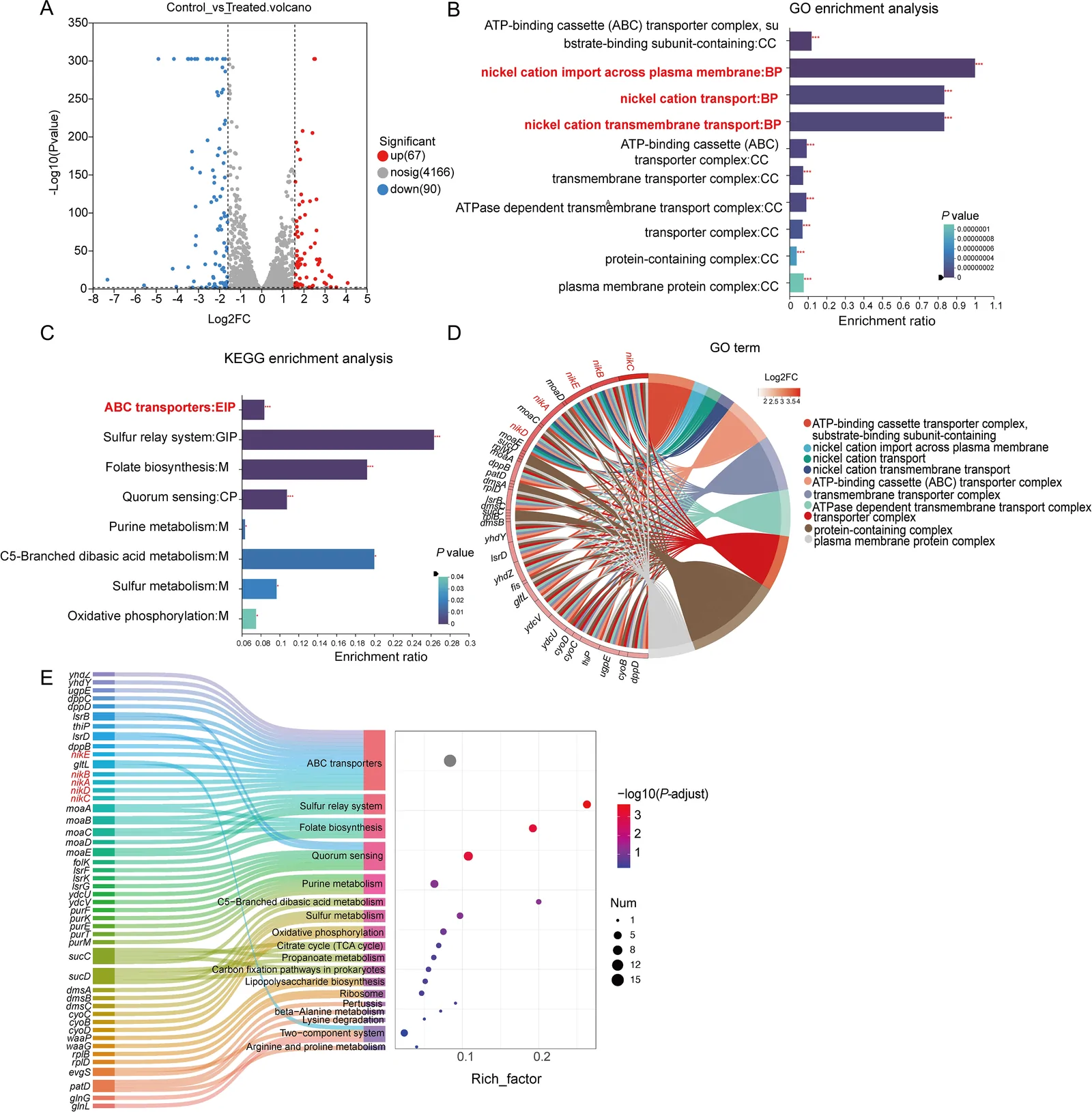
Transcriptomic analysis reveals upregulation of nickel transport pathways in NDM-positive bacteria under L-His treatment. **(A)** Volcano plot of differentially expressed genes (DEGs) in *E. coli* G6 treated with or without L-His (10 mM) (n = 3 per group). **(B)** GO enrichment analysis of DEGs. Processes related to nickel transport are highlighted in red. **(C)** KEGG enrichment analysis of DEGs. ABC transporters pathway is highlighted in red. **(D)** GO enrichment chord diagram of DEGs. The left side is the gene, and the more up the gene, the greater the differential expression multiple. The right side is the GO Term information on the significant enrichment of differential genes. **(E)** Sankey diagram and bubble map of the top 18 KEGG pathways enriched with DEGs. Ni^2+^ transport-related genes, *nikABCDE* is highlighted in red.

RT-qPCR analysis further confirmed the significant upregulation of Ni^2+^ transport-related genes in L-His-treated bacteria (Fig 5A). The NikAB transporter requires L-His as a ligand to facilitate Ni^2+^ transport: one Ni^2+^ forms a complex with two L-His molecules before the complex is recognized and bound by the transporter [23]. We therefore hypothesized that exogenous L-His, acting as a ligand for nickel ions, would promote NikAB-mediated Ni^2+^ uptake and accumulation in bacteria, thereby altering intracellular ion homeostasis and potentially affecting antibiotic susceptibility. To verify this, we constructed Δ*nikAB* knockout and *nikA*-overexpression strains. Bactericidal assays demonstrated that the synergistic bactericidal effect of L-His and MEM was significantly attenuated in the Δ*nikAB* mutant (Fig 5B), while *nikA*-overexpression dramatically enhanced bacterial killing by varying concentrations of MEM (Fig 5C). These results confirmed that *nikABCDE* played a critical role in L-His-mediated resensitization to MEM. Using Inductively Coupled Plasma Mass Spectrometry (ICP-MS), we found that the addition of L-His considerably enhanced the intracellular Ni^2+^ levels (Fig 5D). Similarly, Ni^2+^ concentration was markedly elevated in the *nikA*-overexpressing strain, but remained at comparatively low levels in the Δ*nikAB* mutant, regardless of L-His supplementation (Fig 5E).

**Fig 5:**
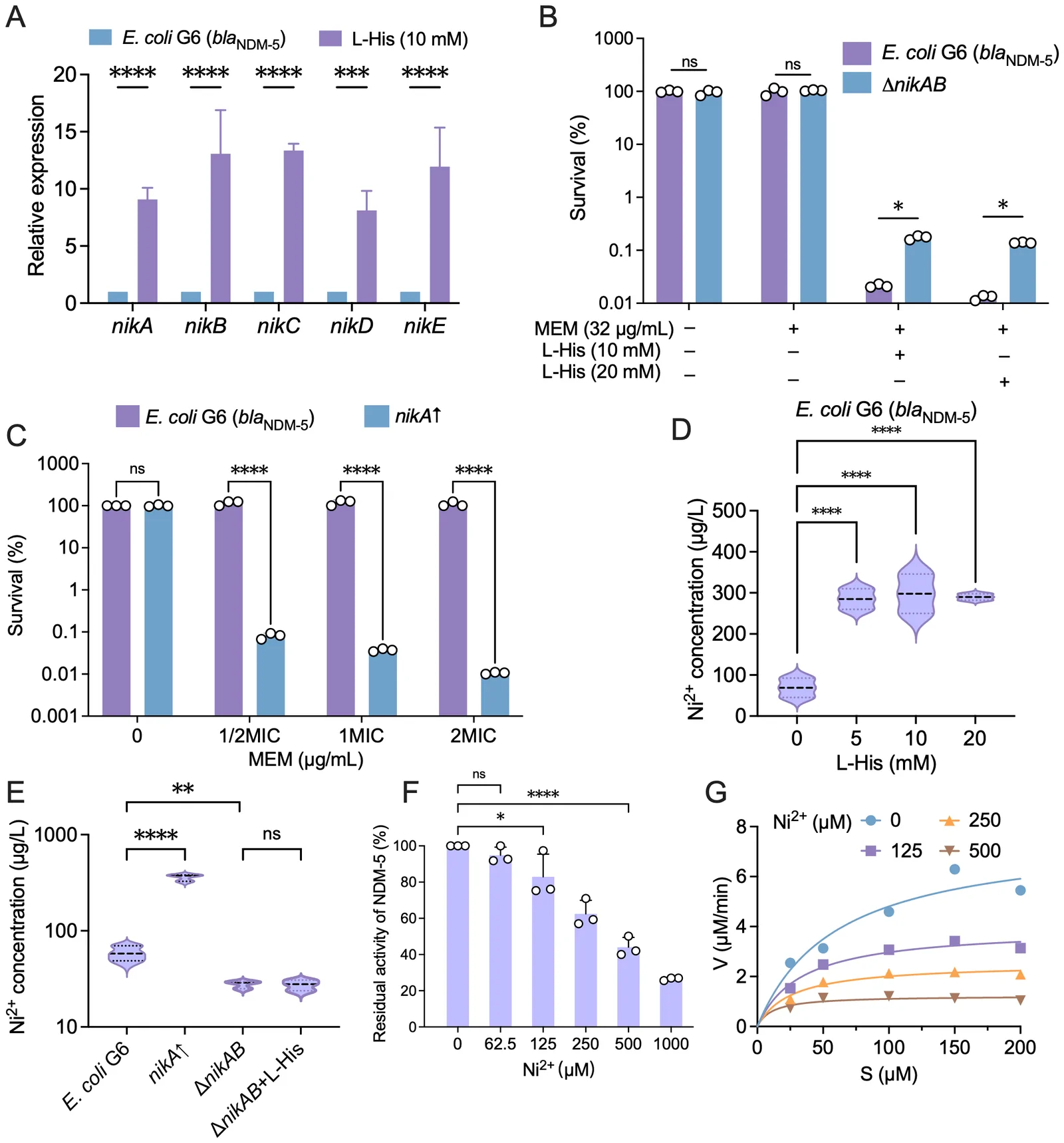
Enhanced nickel transport upon L-histidine exposure contributes to the synergistic effect of L-histidine and MEM. (**A)** Transcriptional levels of nickel transport-related genes (*nikA-E*) in *E. coli* G6 following treatment with 10 mM L-His. **(B)** Bacterial survival of wild-type (*E. coli* G6) and Δ*nikAB* strains after treatment with L-His and/or MEM. **(C)** Survival rate of *E. coli* G6 and *nikA*-overexpression strains exposed to increasing concentrations of MEM. **(D)** Bacterial intracellular Ni^2+^ concentration treated with increasing concentrations of L-His. **(E)** Intracellular nickel levels in wild type, Δ*nikAB*, and *nikA*-overexpression strains with or without L-His (10 mM). **(F)** Hydrolysis activity of NDM-5 against cefalotin in the presence of increasing concentrations of nickel. **(G)** Nonlinear regression analysis of enzyme kinetics with NiCl_2_. The initial reaction velocity (V) was plotted against substrate concentration (S). Data points were fitted (solid lines) to the Michaelis-Menten equation. Inhibitor concentrations were 0–500 µM and the Vmax were 7.689 μM/s, 3.953 μM/s, 2.526 μM/s and 1.231 μM/s. The decreased Vmax indicates non-competitive inhibition. The data from three biological replicates were presented as mean ± SD, and the statistical significance was determined using a nonparametric one-way ANOVA or unpaired *t* test. The levels of significance were denoted as *P < 0.05, **P < 0.01, ***P < 0.001, ****P < 0.0001. ns, not significant.

Given that metal ions such as silver [24], gold [25], and bismuth [26] can inactivate NDM by displacing active-site zinc ions, we sought to test whether Ni^2+^ exerts a similar effect. In *vitro* enzyme activity tests revealed that Ni^2+^ could indeed suppress NDM hydrolytic activity (Fig 5F). Unlike L-His, the enzyme kinetic profile of the Ni^2+^ exhibited distinct characteristics of non-competitive inhibition (Fig 5G). To demonstrate that elevated intracellular Ni^2+^ can enhance the inhibitory effect of antibiotics on bacteria, and that this effect is influenced by the nickel ion transporter and its transport ligand L-His, checkerboard assays and growth curve analysis were performed. The checkerboard assay results showed that Ni^2+^ alone did not synergize with MEM (S5A–S5C Figs), and the addition of trace amounts of L-His (1 mM) only marginally promoted the synergistic effect between Ni^2+^ and MEM (S5D–S5F Fig). However, growth curve analysis indicated that the inhibitory effect of nickel ions and MEM on bacteria was primarily observed during the logarithmic growth phase. Under *nikA* overexpression conditions, the combination of MEM and Ni^2+^ slowed bacterial growth, whereas this effect was not pronounced in wild-type or *nikAB* knockout strains. Furthermore, the inclusion of L-His strengthened this growth-inhibitory effect in the presence of MEM and Ni^2+^, particularly in the *nikA*-overexpressing strain (S5G–S5I Figs). Collectively, these findings indicate that L-His increases intracellular Ni^2+^ levels by promoting high expression of nickel transport genes and acting as a Ni^2+^ transport ligand. The accumulated nickel further inhibits NDM enzyme activity through a non-competitive mechanism, thereby enhancing MEM efficacy against NDM-positive bacteria.

### L-Histidine improves the effectiveness of MEM in *vivo*

To further assess the therapeutic potential of L-His and MEM combination in *vivo*, we utilized a murine peritonitis-sepsis model. Female BALB/c mice were intraperitoneally injected with a lethal dose of *E. coli* G6, and subsequently treated with vehiclecontrol, MEM (20 mg/kg), L-His (150 mg/kg) monotherapy, or their combination. While L-His monotherapy failed to protect any mice, MEM alone only afforded 20% survival at 48 h, the combination therapy significantly improved survival to 70% (Fig 6A).

We further assessed the ability of combination therapy to reduce bacterial burden in mice infected with non-lethal NDM-positive *E. coli* G6. Mice were infected with a sublethal dose of *E. coli* G6 and treated as above. After 12 h, bacterial loads in the liver, spleen, and kidneys were determined using agar plate counting. The combination therapy led to a significant reduction in CFU counts in all three organs compared to either monotherapy (Fig 6B-D). Consistent with improved bacterial clearance, cytokine profiling showed that co-treatment elevated anti-inflammatory cytokines (IL-10 and IL-4) while suppressing pro-inflammatory mediators (IL-1β and IL-6) (Fig 6E-H). Histological sections of liver tissues further supported these findings, revealing marked hepatic pathology in the infected group, characterized by diffuse hepatocellular edema, vacuolization, multifocal necrosis, and extensive cellular infiltration within and around the necrotic areas. In contrast, mice treated with the L-His plus MEM combination showed substantially ameliorated liver injury, as evidenced by improved hepatocellular architecture, reduced cellular infiltration, and fewer foci of parenchymal damage (Fig 6I-L). Together, these in *vivo* results confirm that L-His effectively enhances MEM efficacy against NDM-positive infections, improving survival, reducing bacterial load, and mitigating infection-induced inflammation and tissue injury.

**Fig 6:**
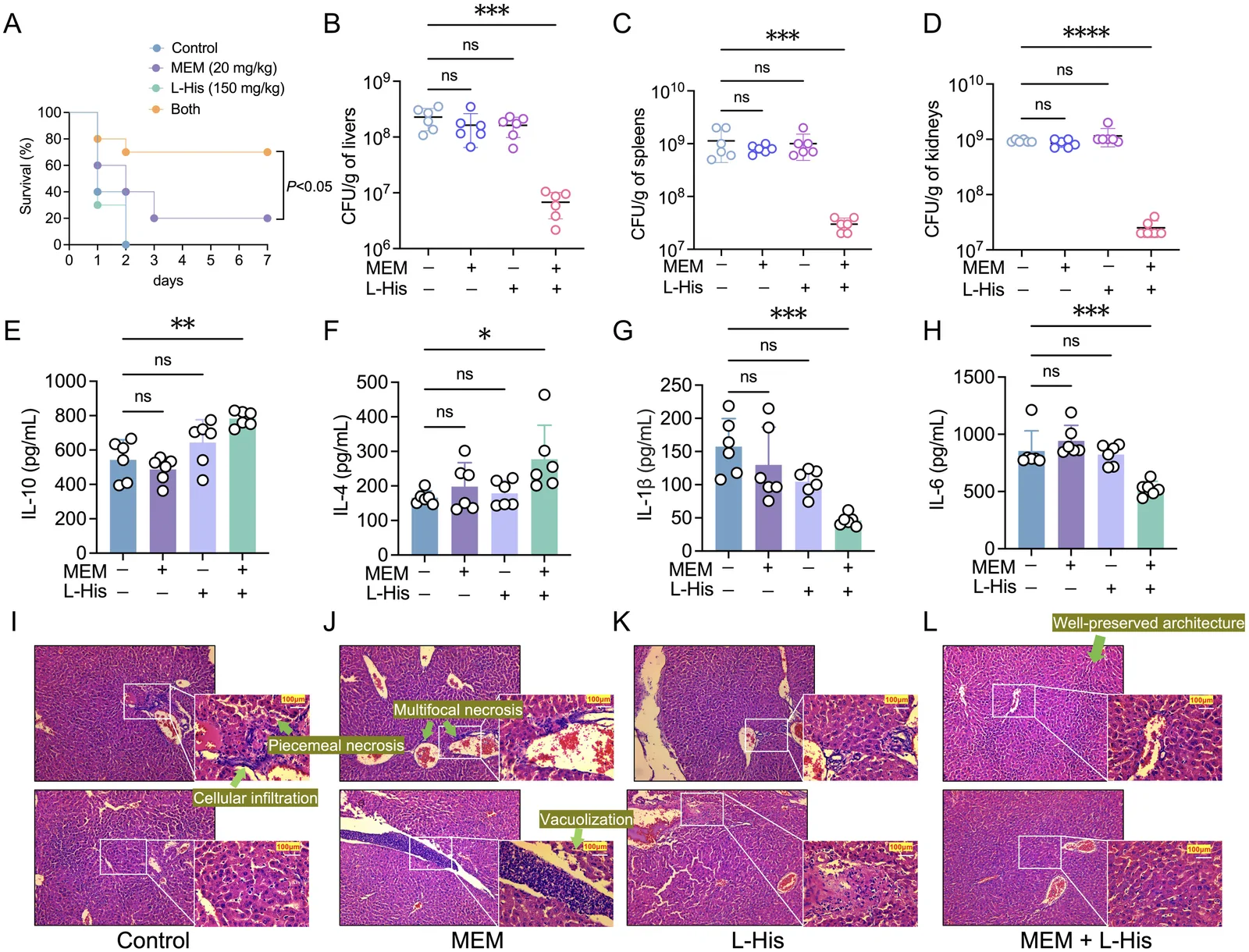
L-Histidine improves the *in* *vivo* effectiveness of MEM. **(A)** Survival curves of Balb/c mice (n = 10 per group) infected intraperitoneally with a lethal dose of *E. coli* G6 (1 × 10^9^ CFU) and treated with a single dose of L-His (150 mg/kg), MEM (20 mg/kg), or their combination. The statistical significance was determined by Mann–Whitney U test. **(B-D)** Bacterial burdens in the liver (B), spleen (C), and kidney (D) of mice (n = 6 per group) infected with a sublethal dose of *E. coli* G6 (1 × 10^8^ CFU) and treated with MEM alone or in combination with L-His. **(E-H)** Serum levels of anti-inflammatory cytokines IL-10 (E) and IL-4 (F), and the pro-inflammatory cytokines IL-1β (G) and IL-6 (H). **(I-L)** Representative H&E-stained liver sections from mice subjected to the indicated treatments. Scale bar, 100 μm. The data were presented as mean ± SD, and the significance was determined by non-parametric one-way ANOVA. *P < 0.05, **P < 0.01, ***P < 0.001, ****P < 0.0001. ns, not significant.

## Discussion

The escalating prevalence of drug-resistant bacterial pathogens underscores the limitations of conventional single-target therapeutic strategies and highlights the urgent need for innovative strategies to combat AMR [1]. One promising avenue involves modulating bacterial metabolic pathways to restore antibiotic sensitivity in resistant strains. In this study, we combined untargeted metabolomics with functional screening to identify L-His as a potent antibiotic enhancer capable of resensitizing NDM-positive bacteria to β-lactam antibiotics. The combination of L-His and MEM demonstrated great *in vivo* effectiveness while displaying a favorable safety profile. Mechanistically, we demonstrated that L‑His counteracts NDM‑mediated resistance through two complementary pathways (Fig 7). First, L‑His acts as a direct NDM inhibitor by competing for and chelating the active-site zinc ions, thereby limiting NDM activity and preventing β-lactam hydrolysis. Second, L‑His upregulates the *nikABCDE* operon and serves as a nickel‑transport ligand, promoting intracellular accumulation of Ni^2+^, which in turn inhibits NDM activity via a non‑competitive mechanism and further safeguards internalized antibiotics from degradation. It should be noted that the untargeted metabolomics analysis in our discovery-driven screening strategy served as a hypothesis-generating tool to nominate candidate metabolites for functional validation, rather than as principal evidence supporting the study’s conclusions.

**Fig 7:**
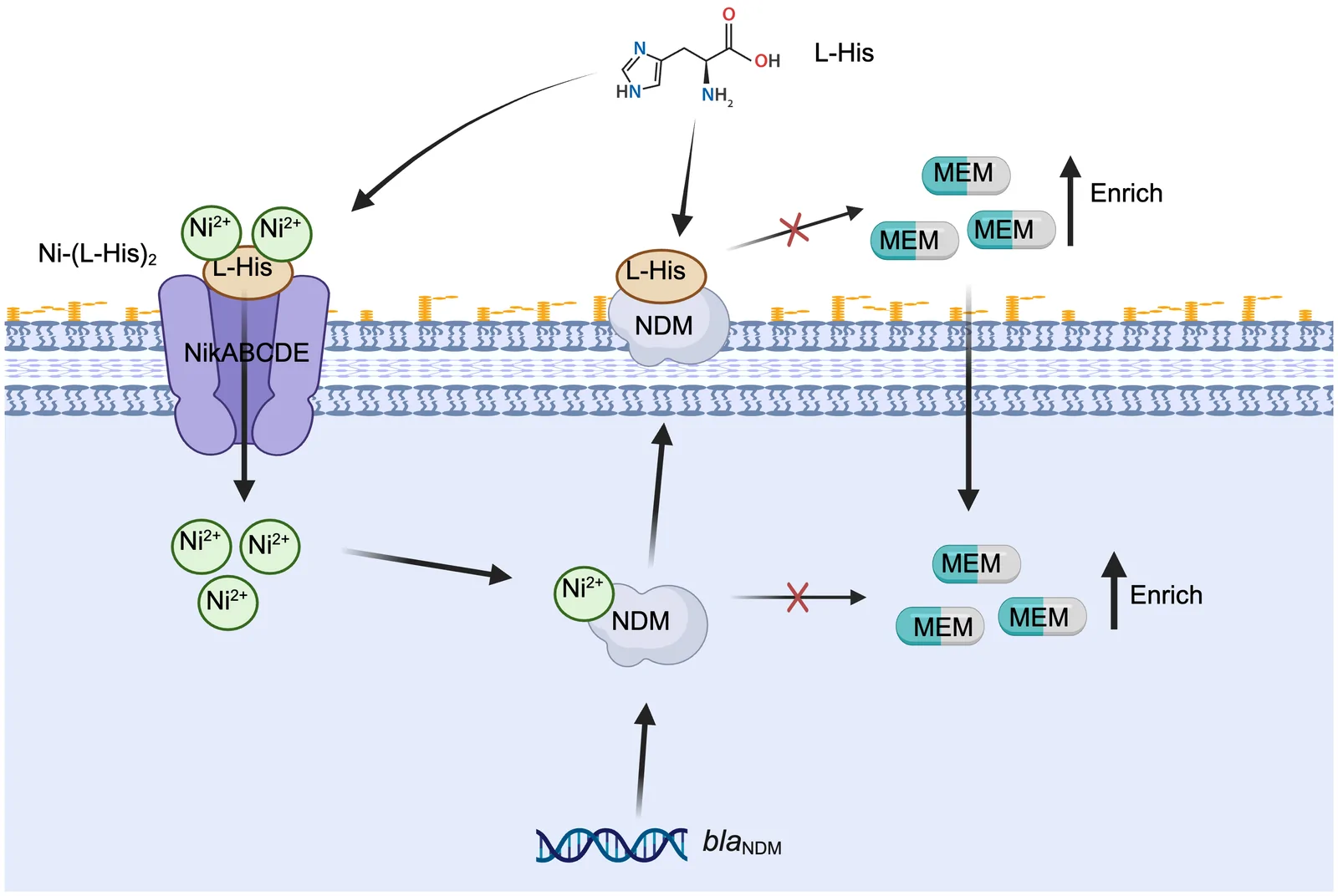
Schematic illustration of the underlying mechanism of L-histidine restoring the antibacterial activity of MEM against NDM-positive bacteria. L-His inhibits NDM enzymes from hydrolysing antibiotics. In addition, it upregulates the expression of nickel ion transport-related genes to boost cellular Ni^2+^ influx and Ni^2+^ further suppresses NDM-mediated MEM hydrolysis. L-His, L-Histidine; NDM, New Delhi metallo-β-lactamase; MEM, meropenem.

Importantly, the synergistic activity of L-His with meropenem (MEM) was consistently observed across NDM-1- and NDM-5-producing strains, spanning both engineered and clinical isolates. Although these variants share > 90% sequence identity and both belong to subclass B1 [27], they differ at several active-site residues that could affect inhibitor binding. The preservation of L-His activity against both enzymes therefore indicates that its dual mechanism of action, zinc chelation coupled with nickel-mediated inhibition, targets structural features conserved within the NDM family. More notably, L-His also restored MEM susceptibility in IMP-producing clinical isolates; IMP is a class B metallo-β-lactamase (MBL) sharing only ~40% sequence identity with NDM-1 and possessing a distinctly different active-site architecture [28]. This cross-enzyme activity strongly suggests that the mode of action of L-His is not NDM-specific, but instead targets conserved functional elements across the broader class B MBL family, thereby substantially enhancing the translational relevance of our findings for combating diverse MBL-producing pathogens.

Previous efforts have identified several MBL inhibitors through various approaches [29,30]. In 2014, King et al. identified the fungal natural product aspergillomarasmine A (AMA) through a cell-based screening approach, demonstrating its potent inhibitory activity against both NDM-1 and VIM-2 metallo-β-lactamases [31]. Subsequent research by Chiou et al. revealed that ebselen, an antioxidant compound previously established as safe for human use, may serve as a promising NDM-1 inhibitor through its targeting of cysteine residues in the enzyme’s active site [32]. In another study, Klingler et al. evaluated eleven clinically approved thiol-containing drugs, identifying four (captopril, tiopronin, dimercaprol, and thiorphan) with inhibitory activity against NDM-1, VIM-1, and IMP-7 [33]. Despite these ongoing efforts, no NDM inhibitors were approved for clinical use owing to unfavorable pharmacological properties and potential toxic effects. In this context, L-His represents a distinctive candidate with inherent biocompatibility and established safety profiles in *vivo*.

Although several amino acids have been identified to exhibit synergistic effects with antibiotics [22,34,35], the direct inhibitory effect on resistance enzymes has not been previously documented. L-His, an essential amino acid, plays multifaceted physiological roles including proton buffering, metal ion chelation, scavenging of reactive oxygen and nitrogen species, erythropoiesis, and modulation of the histaminergic system [36]. Notably, the tolerable upper intake level of L-His in healthy adults remains undefined, with estimated safe ranges potentially spanning 4.5-24 g/day [37]. In our study, the maximum administered dose of 150 mg/kg was determined to be within established safety parameters based on current evidence, supporting its translational potential.

While the role of L-His as a nickel transport ligand has been established [23], our discovery of its ability to increase intracellular Ni^2+^ levels and subsequently inhibit NDM represents a novel anti-infective strategy. This conclusion was supported by the significant reduction of synergy upon *nikAB* deletion. Many metal ions possess intrinsic antibacterial properties or can potentiate antibiotic activity. For instance, gallium disrupts iron metabolism to treat pulmonary infections [38], while gold [25] and silver [24] ions inhibit MBLs by displacing zinc cofactors. Also, bismuth enhances *Pseudomonas aeruginosa* susceptibility to antibiotics by perturbing iron homeostasis [26]. Unlike conventional heavy metal antimicrobials, Nn^2+^ has not been previously reported to exert such effects and this way leverages physiological nickel transport pathways. Given the trace physiological levels of Ni² ⁺ , this approach may minimize systemic toxicity risks while effectively countering resistance. Furthermore, the Ni^2+^-enriching effect of L-His prompts a reconsideration of the therapeutic potential of metal ions in combating bacterial infections.

In conclusion, our study reveals that exogenous L‑histidine supplementation metabolically reprograms NDM‑positive pathogens, effectively restoring MEM susceptibility through a dual mechanism that combines direct enzyme inhibition with nickel‑dependent NDM suppression. Moreover, the activity against IMP-producing strains indicates that this strategy may extend beyond NDM to combat a broader spectrum of class B MBL-mediated carbapenem resistance. The therapeutic efficacy of this combination strategy has been validated in animal infection models. Nevertheless, further investigations are warranted to fully assess the clinical feasibility of L‑His as an adjunctive therapy. Collectively, our findings establish L-His as a promising multi-mechanistic antibiotic adjuvant against life-threatening infections caused by NDM-producing multidrug-resistant pathogens, with potential extension to other clinically relevant MBL-positive pathogens.

## Materials and methods

### Ethics statement

All animal experiments were approved by the Animal Care Committee of Yangzhou University and conducted in accordance with the guidelines for Laboratory Animal Welfare and Ethical of the Jiangsu Administrative Committee of Laboratory Animals (SYXK-2022–0044).

### Bacterial strains and reagents

All the strains used in this study and their corresponding MICs were listed in S1 Table. Briefly, a single colony of each strain was inoculated in fresh Luria Bertani broth (LB, Qingdao Hope Bio-technology) and incubated overnight at 37 °C with shaking at 200 rpm. Then cultures were diluted 1:100 in 30 mL of fresh LB broth in 50 mL flasks and grown to the exponential phase. Bacterial cells were harvested by centrifugation at 4,000 g for 7 min, washed with sterile saline, and resuspended in M9 minimal medium (Na_2_HPO_4_ 6.78 g/L, KH_2_PO_4_ 3.0 g/L, NaCl 0.5 g/L, NH_4_Cl 1.0 g/L, MgSO_4_ 0.241 g/L, CaCl_2_ 0.011 g/L, glucose 4 g/L, pH = 6.8) to an OD_600_ of 0.5. Antibiotics or metabolites were added at specified concentrations. Collected cells were used for subsequent plate counting or biochemical analysis. Antibiotics were obtained from the China Institute of Veterinary Drug Control, all other chemical reagents were purchased from Yuan Ye, Solarbio or Aladdin. M9 minimal medium was purchased from Coolaber company (SL0060).

### MICs determination

The minimum inhibitory concentrations (MICs) of antibiotics were determined using the broth microdilution method in Mueller-Hinton broth (MHB), with serial two-fold dilutions of each drug. After 18 h of incubation at 37°C, MIC values were recorded as the lowest concentration that completely inhibited visible bacterial growth, in accordance with CLSI 2021 guidelines.

### Metabolomics profiling

*E. coli* DH5α (pUC19) and *E. coli* DH5α (pUC19-*bla*_NDM-5_) were cultured in 1.5 mL of fresh LB broth without antibiotics to the exponential phase (6 h). Metabolites were extracted and analyzed by liquid chromatography-mass spectrometry (LC-MS). Raw data were processed and matched against the HMDB and Majorbio databases for metabolite identification. Differential metabolites were annotated and mapped to KEGG pathways (https://www.kegg.jp/kegg/pathway.html). Pathway enrichment analysis was performed using the scipy.stats package in Python, with significance assessed by Fisher’s exact test.

### Transcriptomic analysis

Overnight cultures of *E. coli* G6 were diluted 1:100 in fresh MH broth and grown to mid-exponential phase (4 h, 37°C, 200 rpm). Cells were collected and washed 3 times with PBS and then resuspended to 10^5^ CFU/ml. L-His (10 mM) was added, and cultures were incubated for 4 h under the same conditions. Total RNA was extracted using the EASYspin Plus kit, and quality was assessed using a NanoDrop 2000, agarose gel electrophoresis, and an Agilent 2100 Bioanalyzer. Libraries were constructed with the TruSeq Stranded Total RNA Library Prep Kit and sequenced. Reads were aligned to a reference genome, and gene expression quantification was performed with RSEM. Differential expression analysis was conducted using DESeq2, and functional annotation was performed using GO and KEGG databases.

### Checkerboard assays

Synergy was assessed using checkerboard assays in 96-well plates with two-fold serial dilutions of antibiotics and metabolites (8 × 8 matrix). Bacterial suspensions (1.0 × 10^6^ CFU/well) were co-incubated with compounds for 18 h at 37°C. Absorbance at 600 nm was measured, and the fractional inhibitory concentration index (FICI) was calculated as follows: FIC index = FICIa + FICIb = MICab/ MICa + MICba/MICb. Experiments were performed in triplicate.

### Growth curves measurement

Overnight cultures were diluted 1:1,000 in fresh LB broth and exposed to different treatments in 96-well plates (final volume 200 µL). Mineral oil (50 μL) was added to prevent evaporation. OD_600_ was monitored continuously using the Infinite E Plex Microplate reader (Tecan).

### Effect of L-His on antibiotic killing

Overnight cultures of NDM-positive strains were diluted 1:100 in fresh MHB medium and grown to exponential phase (4 h, 37°C, 200 rpm). Cells were washed with PBS, resuspended in M9 medium (Sangon Biotech, Shanghai, China) and treated with drugs for 4 hours. Bacterial survival was assessed by serial dilution and spot plating on LB agar. Survival rates were normalized to the untreated control.

### Time-kill assays

NDM-positive bacteria were grown to logarithmic phase, washed, and resuspended in M9 medium (OD_600_ = 0.5). Cultures were treated with MEM (1/2 MIC), L-His (10 mM), or the combination. Aliquots were collected at 0, 4, 8, 24, and 48 hours, serially diluted, and plated for CFU counting. Data from three independent trials were plotted as log_10_ CFU/mL versus time.

### Resistance development study

*E. coli* G6 was passaged for 12 generations in LB containing subinhibitory concentrations of MEM and/or L-His. Cultures were incubated at 37°C with shaking (200 rpm) for 12 h per passage. MICs of evolved populations were determined after each passage.

### Enzyme inhibition assays

NDM-5 activity was measured using nitrocefin as a substrate in 96-well plates. Absorbance was read at 492 nm using a microplate reader (Tecan). Positive controls were performed in the presence of enzyme and in the absence of inhibitors, whereas negative controls lacked enzyme. Residual activity = [(A − A0) / (A100 − A0)] × 100%, where A is the absorbance of the test well, and A0 and A100 represent 0% and 100% activity as determined in the negative controls and positive controls, respectively.

### LC-MS analysis

Bacterial suspensions with or without L-His (20 mM) were incubated with MEM at different concentrations (16, 32, and 64 µg/mL) for 1.5 h. Supernatants were collected by centrifugation (10,000 rpm, 20 min), filtered (0.2 µm), and analyzed by LC-MS. Separation used a C18 column (4.6 × 250 mm) with mobile phase A (10 mM KH_2_PO_4_) and B (acetonitrile). The gradient elution program was as follows: 0–0.5 min, 99% A; 0.5–1.5 min, 99–70% A; 1.5–2.0 min, 70% A; 2.0–3.0 min, 70–10% A; 3.0–3.5 min, 10% A; 3.5-3.6 min, 10–99% A; and 3.6–5.0 min, 99% A. Injection volume: 5 μL. MEM was detected using positive electrospray ionization and multiple reaction monitoring.

### Construction of knockout strain

The upstream and downstream homology arms of the target gene *nikAB* were amplified from *E. coli* G6 genomic DNA using primers listed in S3 Table. Products were ligated into the linearized pRE112 plasmid and transfected into *E. coli* DH5α-λ receptor cells. Plasmid DNA was isolated and electroporated into *E. coli* X7213. Conjugation with G6 was performed on plates containing 2,6-diaminopimelic acid (DAP). Transconjugants were selected on sucrose plates and verified by PCR and sequencing.

### Construction of gene overexpression strain

The *nikA* gene was amplified from G6 genomic DNA using primers in S3 Table and cloned into the pBAD44 plasmid. The construct was transformed into *E. coli* DH5α, verified by sequencing, and then introduced into G6. Overexpression was confirmed by PCR and sequencing. Expression was induced by adding L-arabinose to a final concentration of 0.2% when the culture reached an OD_600_ of 0.5.

### ICP-MS analysis of nickel uptake

Bacteria were grown to exponential phase, resuspended in M9 medium (OD_600_ = 0.5), and treated with L-His (0, 5, 10, or 20 mM) for 4 h. Cells were washed with PBS, digested with 68% HNO_3_ at 60 °C overnight, and analyzed by ICP-MS (Elan DRC-e) using ^115^In as an internal standard.

### RT-qPCR analysis

RNA was extracted using Bacteria RNA Extraction Kit (Vazyme) and reverse-transcribed with PrimeScript RT Kit and gDNA Eraser (Takara, Dalian, China), following the provided instructions. RT-qPCR analysis was performed on a 7500 Fast Real-Time PCR System (Applied Biosystem, CA, USA) using the ChamQ SYBR Color qPCR Master Mix (Vazyme). Primers are listed in S3 Table. A 2-step PCR amplification standard procedure was used for thermal cycling (40 cycles of 95 °C for 30 s, 95 °C for 10 s, and 60 °C for 30 s). Based on the 2^−ΔΔCt^ method, the fold change of mRNA expression relative to a reference gene (16S rRNA) was calculated.

### L-His quantification

Bacterial strains were grown overnight, subcultured 1:100 into fresh LB, and incubated to mid-log phase (~4 h). Cells were harvested by centrifugation, washed with PBS, and lysed by ultrasonication on ice. After centrifugation, supernatants were collected. L-his levels were measured using a competitive ELISA kit (JingMei, 0298Z2), following the manufacturer’s protocol.

### Animal infection models

For survival studies, six-week-old female BALB/c mice (n = 10 per group) were infected intraperitoneally with *E. coli* G6 (1 × 10^9^ CFU). Treatments (L-His 150 mg/kg, MEM 20 mg/kg, or combination) were administered 4 h post-infection, and survival was monitored for 7 days.

For bacterial load, cytokine, and histology analyses, mice (n = 6 per group) were infected with a sublethal dose (1 × 10^8^ CFU) and treated as above. Mice were euthanized at 12 h post-infection; organs were harvested for CFU counting, blood was collected for cytokine ELISA, and liver tissues were fixed for H&E staining.

### Statistical analyses

Statistical analysis was conducted using GraphPad Prism Version 9.0. All data were obtained from a minimum of three biological replicates. For the in *vitro* studies, the statistical significance was determined through an unpaired t test between 2 groups or one-way analysis of variance (ANOVA) among multiple groups. For the in *vivo* studies, log-rank (Mantel–Cox) test or the Mann-Whitney U test was used to calculate *P* values. Significance levels are indicated by asterisks as follows: ns, not significant; *P < 0.05, **P < 0.01, ***P < 0.001, ****P < 0.0001

## Supporting information

S1 Fig KEGG pathway enrichment network diagram of DEMs. Yellow square and green circular nodes represent metabolites and KEGG pathways, respectively. Pathway node size corresponds to the number of associated DEMs. Amino acid metabolites are highlighted in red.
(TIF)

S2 Fig Synergistic antibacterial activity of L-His with MEM against NDM-positive bacteria. (A) L-His levels in NDM-positive and NDM-negative strains. (B) Bacterial survival of *E. coli* G6 (*bla*_NDM-5_) following treatment with MEM (1/2 MIC) in combination with different amino acids (30 mM). (C) Bactericidal activity of L-His (30 mM) in combination with the indicated β-lactam antibiotics against *E. coli* C3(*bla*_NDM-1_). CTX: cefotaxime, CAZ: ceftazidime, CRO: ceftriaxone, MEM: meropenem, IPM: imipenem, AMX: amoxicillin, AMP: ampicillin, TIC: ticarcillin, PIP: piperacillin, CLO: cloxacillin. (D-F) Growth curves of various NDM-positive strains treated with L-His (30 mM) and/or MEM (1/2 MIC). (G-I) Dose-dependent bactericidal effects of L-His in conjunction with MEM against NDM-positive strains. Experiments were performed with three biological replicates and data are means ± SD, and the statistical significance was determined by one-way ANOVA or unpaired *t*-test. *P < 0.05, **P < 0.01, ***P < 0.001, ****P < 0.0001. ns, not significant.
(TIF)

S3 Fig Checkerboard assay of L-His and MEM against bacteria carrying *bla*_KPC-2_ or *bla*_IMP-4_. (TIF)

S4 Fig PCA plot of transcriptomic results for *E. coli* G6 (*bla*_NDM-5_) treated with or without L-His (10 mM) addition. PCA of transcriptomics, an unsupervised PCA plot was applied to examine the clustering characteristics of different genes. Colored dots represent individual samples (n = 3).
(TIF)

S5 Fig Synergistic effect of MEM and Ni^2+^. (A-C) Checkerboard assays evaluating the interaction between Ni^2+^ and meropenem against G6, Δ*nikAB*, and *nikA*-overexpression strains. (D-F) Checkerboard assays evaluating the interaction between Ni^2+^ and MEM against *E. coli* G6, *nikA*-overexpression, and Δ*nikAB* strains with the addition of L-His (1mM). (G-I) Growth curves of G6, *nikA*-overexpression, and Δ*nikAB* strains under the treatment with MEM alone, MEM + Ni^2+^, MEM + L-His (1mM), or the triple combination of MEM + Ni^2+^+L-His. Experiments were performed with three biological replicates and data are means ± SD, and the statistical significance was determined by one-way ANOVA. **P < 0.01, ***P < 0.001, ****P < 0.0001. ns, not significant.
(TIF)

S1 Table MICs of MEM against the tested strains in the presence and absence of L-His (μg/mL). (XLSX)

S2 Table Antimicrobial susceptibility of *E. coli* C3 (*bla*_NDM-1_). (XLSX)

S3 Table Primer sequences used in this study. (XLSX)
